# Correction: Highly efficient degradation of reactive black KN-B dye by ultraviolet light responsive ZIF-8 photocatalysts with different morphologies

**DOI:** 10.1039/d3ra90020g

**Published:** 2023-03-23

**Authors:** Le Gia Trung, Minh Kim Nguyen, Thi Dieu Hang Nguyen, Vy Anh Tran, Jin Seog Gwag, Nguyen Tien Tran

**Affiliations:** a Department of Physics, Yeungnam University Gyeongsan Gyeongbuk 38541 Republic of Korea sweat3000@ynu.ac.kr; b The University of Da Nang, University of Technology and Education 48 Cao Thang St., Hai Chau Dist. Da Nang City 550000 Vietnam; c The University of Da Nang, University of Science and Technology (DUT) 54 Nguyen Luong Bang Da Nang 550000 Vietnam; d Institute of Applied Technology and Sustainable Development, Nguyen Tat Thanh University Ho Chi Minh City 700000 Vietnam; e Faculty of Environmental and Food Engineering, Nguyen Tat Thanh University Ho Chi Minh City 700000 Vietnam; f Center for Advanced Chemistry, Institute of Research and Development, Duy Tan University 03 Quang Trung Da Nang 550000 Vietnam trannguyentien@duytan.edu.vn; g Faculty of Natural Sciences, Duy Tan University 03 Quang Trung Da Nang 550000 Vietnam

## Abstract

Correction for ‘Highly efficient degradation of reactive black KN-B dye by ultraviolet light responsive ZIF-8 photocatalysts with different morphologies’ by Le Gia Trung *et al.*, *RSC Adv.*, 2023, **13**, 5908–5924, http://dx.doi.org/10.1039/d2ra08312d.

The authors regret that the one of the affiliations (affiliation b) was incorrectly shown in the original manuscript. The corrected list of affiliations is as shown above.

The Royal Society of Chemistry apologises for these errors and any consequent inconvenience to authors and readers.

## Supplementary Material

